# Impact of aging on neck vasculature and brain biochemistry in female mice

**DOI:** 10.14814/phy2.71059

**Published:** 2026-08-30

**Authors:** Allison R. Jones, Amin Jarrahi, Kylee Karpowich, EllaGrey Moody, Lily Renner, Lindsay P. Brown, Caitlin M. Tressler, A. Colleen Crouch

**Affiliations:** ^1^ Department of Biomedical Engineering University of Tennessee Knoxville Tennessee USA; ^2^ Department of Psychology University of Tennessee Knoxville Tennessee USA; ^3^ Department of Biochemistry & Cellular and Molecular Biology University of Tennessee Knoxville Tennessee USA; ^4^ Department of Chemistry University of Tennessee Knoxville Tennessee USA; ^5^ The Johns Hopkins University Applied Imaging Mass Spectrometry Core and Service Center, Division of Cancer Imaging Research, The Russell H. Morgan Department of Radiology and Radiological Science The Johns Hopkins University School of Medicine Baltimore Maryland USA; ^6^ The Sidney Kimmel Comprehensive Cancer Center The Johns Hopkins University School of Medicine Baltimore Maryland USA

**Keywords:** aging in female mice, brain lipidomics, MALDI mass spectrometry imaging, neck vascular biomechanics, neurovascular aging, wall shear stress

## Abstract

Age‐related changes in large‐vessel biomechanics may contribute to neurovascular dysfunction and region‐specific biochemical remodeling in the brain, with important implications for female health. This study investigated the impact of aging on neck vasculature and brain lipid biochemistry in female C57BL/6NHsd mice by comparing young (12 weeks; *n* = 10) and middle‐aged (52 weeks; *n* = 10) cohorts using ultrasound imaging, histology, and mass spectrometry. In vivo ultrasound quantified carotid and jugular hemodynamics, including wall shear stress (WSS), circumferential cyclic strain (CCS), pulsatility index, and volumetric flow. Ex vivo assessments included spatial lipid mapping in vessels and brain, and histology staining to quantify elastin‐to‐collagen ratios. Middle‐aged females exhibited reduced carotid systolic velocity, systolic WSS, and body weight–normalized carotid volumetric flow compared to young controls, while pulsatility index and CCS showed non‐significant decreases. Histology revealed a reduction in the carotid elastin‐to‐collagen ratio, consistent with vascular remodeling. Lipidomic profiling identified age‐dependent shifts in lipid headgroups, including decreases across multiple brain lipid classes, increased N‐acylethanolamides, alongside increases in carotid cardiolipin and lysophosphatidylethanolamine. Hippocampal spectra showed clearer age‐related separation than whole‐brain analyses. Correlation analyses identified moderate to strong associations between vascular biomechanics and lipid features across tissues, supporting coordinated neurovascular and biochemical aging in female mice.

## 1 INTRODUCTION

Aging alters the structure and function of the vascular system in nearly all regions of the body (Donato et al., 2018; Mitchell, 2008). Age‐related changes in our vascular system, particularly the neck vasculature, can affect the structure and functions of the blood–brain barrier (BBB) and the brain (Faraci & Heistad, 1990; Roberts et al., 2018; Walker et al., 2015). Vascular dysfunction manifests in many ways including arterial stiffening, which causes an increase in pulsatile energy reaching the microvasculature. Properly functioning large arteries dampen the pulsatile pressure generated from the ejection of blood from the heart during systole (Chirinos, 2022). Without the ability to dampen this pressure, the microvasculature surrounding the brain may be subjected to higher pressures than it can withstand. Over time, this can lead to damage of the microvasculature, BBB, and possible changes within the brain (Winder et al., 2021). Both vascular stiffening (Donato et al., 2018) and decline in brain function are associated with aging. Although aging in both sexes leads to increased vascular stiffening and neurological decline (Vatner et al., 2021), women have a higher incidence of cardiovascular disease and many neurological diseases after menopause compared to age‐matched men. The connection between arterial stiffness and mortality is almost twofold higher in women versus men (DuPont et al., 2019), and neurological diseases associated with cognitive decline have been shown to affect more women than men with significant age‐related decline and greater deterioration of cognition in females compared to elderly males (Li & Singh, 2014).

A hallmark of age‐related vascular stiffening is an increase in the ratio between collagen and elastin (Kohn et al., 2015). Elastin and collagen allow for the vessels to stretch and recoil to control the pressure of the blood before it reaches the microvasculature. The elastin fibers are responsible for the stretching component of the arteries and have an extremely low rate of replenishment (Baumann et al., 2021). The deficiency of elastin fibers causes a buildup of collagen fibers, which are responsible for returning the artery back to normal state and thus are very stiff and strong (Vatner et al., 2021). The ratio between these fiber components can be analyzed with different staining techniques and visualized with a microscope. Another way to assess vascular dysfunction is to analyze the wall shear stress (WSS) and circumferential cyclic strain (CCS) of the common carotid artery. Lower values of WSS and CCS indicate the existence of vascular dysfunction (Lin et al., 2008). In addition to proteins, lipids have also been implicated in cardiovascular disease (CVD).

Although blood lipids (low‐density lipoprotein cholesterol and triglycerides) have been comprehensively studied in CVD (Liu et al., 2015), the characterization of lipid distribution in the vasculature has been limited with a focus primarily on atherosclerosis (Moerman et al., 2021). Lipid analyses are generally conducted using a combination of liquid chromatography mass spectrometry (LC–MS) and matrix assisted laser desorption ionization mass spectrometry imaging (MALDI MSI) (Xu et al., 2018). MALDI MSI is an analytical technique specifically designed to image the spatial distribution of lipids in tissues.

Not only are lipids relevant in vascular dysfunction, but the brain is also one of the most lipid‐rich organs (Kao et al., 2020) which makes understanding the lipid changes involved in aging vital for understanding the structural and functional changes occurring in the brain. Aging influences the lipid composition in the brain, causing a gradual decrease of certain cerebral lipids after the age of 50. Phospholipids are common in the brain and are responsible for protecting cells from damage. With age, fatty acid metabolism has been shown to change, which indicates that phospholipids relate to age‐related diseases and disorders (Kelley, 2022). Sphingolipids are primarily membrane lipids and are key components of myelin stability, which maintains protection of the nerves. Low numbers of sphingolipids indicate neuronal death, which can lead to age‐related decline. Cholesterol is the main component of myelin and is synthesized in the central nervous system (CNS) shortly after birth (Alaamery et al., 2020; Giussani et al., 2021; Li & Kim, 2021; Lu et al., 2022; Park et al., 2020; Poitelon et al., 2020). Because lipids play an important role in neurodegeneration and neuroinflammation, analyzing the lipid composition may give more insight into the effect of aging on the brain (Jones et al., 2026) (van Kruining et al., 2020).

In this work, ultrasound imaging, histology, and mass spectrometry are used to investigate the impact of aging on vascular composition and function, as well as brain biochemistry, in female mice. Ultrasound imaging is used for in vivo measurements of CCS and WSS in the carotid arteries and jugular veins (Mitchell et al., 2023). MALDI MSI is used to analyze lipid distributions in the carotid artery and jugular vein to identify signatures associated with elevated triglyceride levels and arterial stiffening. These findings are compared with in vivo assessments of vascular function obtained via ultrasound. This work assesses whether age‐related differences in CCS and WSS in the carotid arteries correlate with alterations in vascular lipid composition, elastin‐to‐collagen ratios, and lipid adaptations in the brain. These analyses provide baseline knowledge to support future mechanistic studies of vascular dysfunction and brain pathologies.

## 2 MATERIALS AND METHODS

**FIGURE 1:**
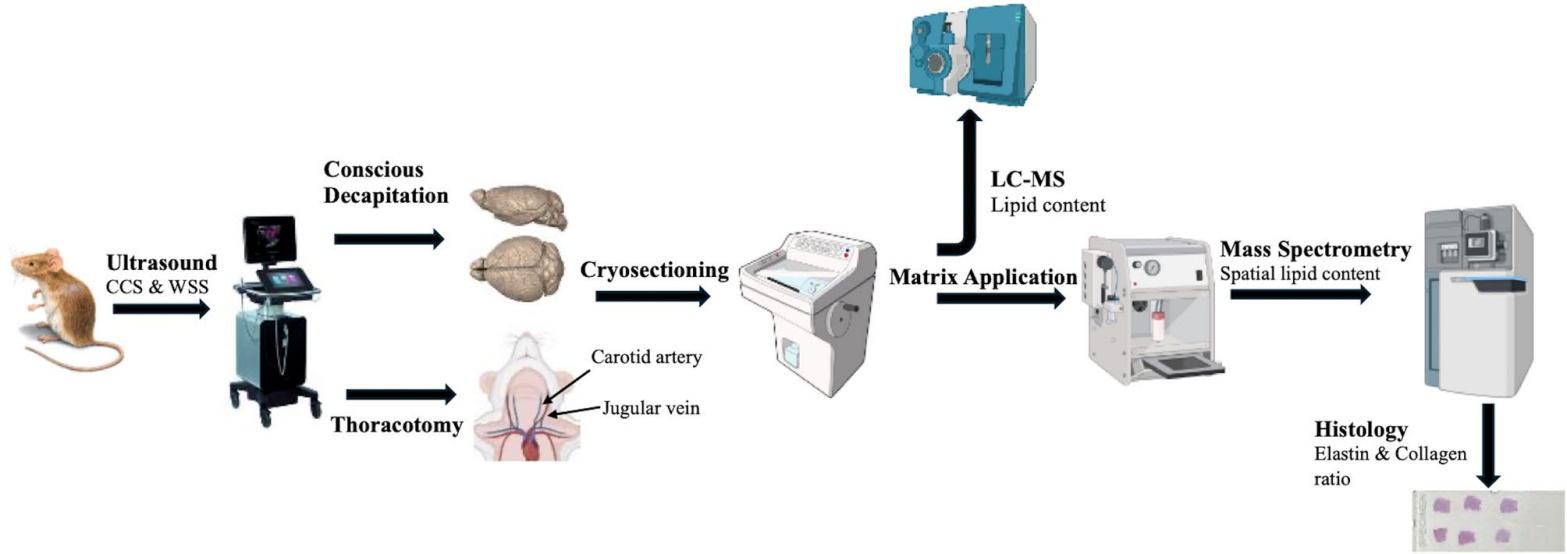
Methods development for research design.

All animal work was approved by the University of Tennessee Institutional Animal Care and Use Committee (IACUC). Healthy C57Bl/6NHsd (Envigo) were used for this experiment. Animals were housed in a room with temperature and humidity maintained to 30%–70% humidity/70–74°F and an alternate 12 h light/dark cycle. Mice were fed Teklad Global 18% Protein Rodent Diet (Product Code: T.2018.15, Envigo). Young (12 weeks, *n* = 10) and middle‐aged (52 weeks, *n* = 10) females were weighed and then imaged via ultrasound (Vevo 3100, Visual Sonics) using previously described methods (Jones et al., 2026). After ultrasound, the neck vasculature and brains were removed for further analysis with mass spectrometry (Jones et al., 2026) and histology. All methods are depicted in Figure 1.

### 2.1 Ultrasound

**FIGURE 2:**
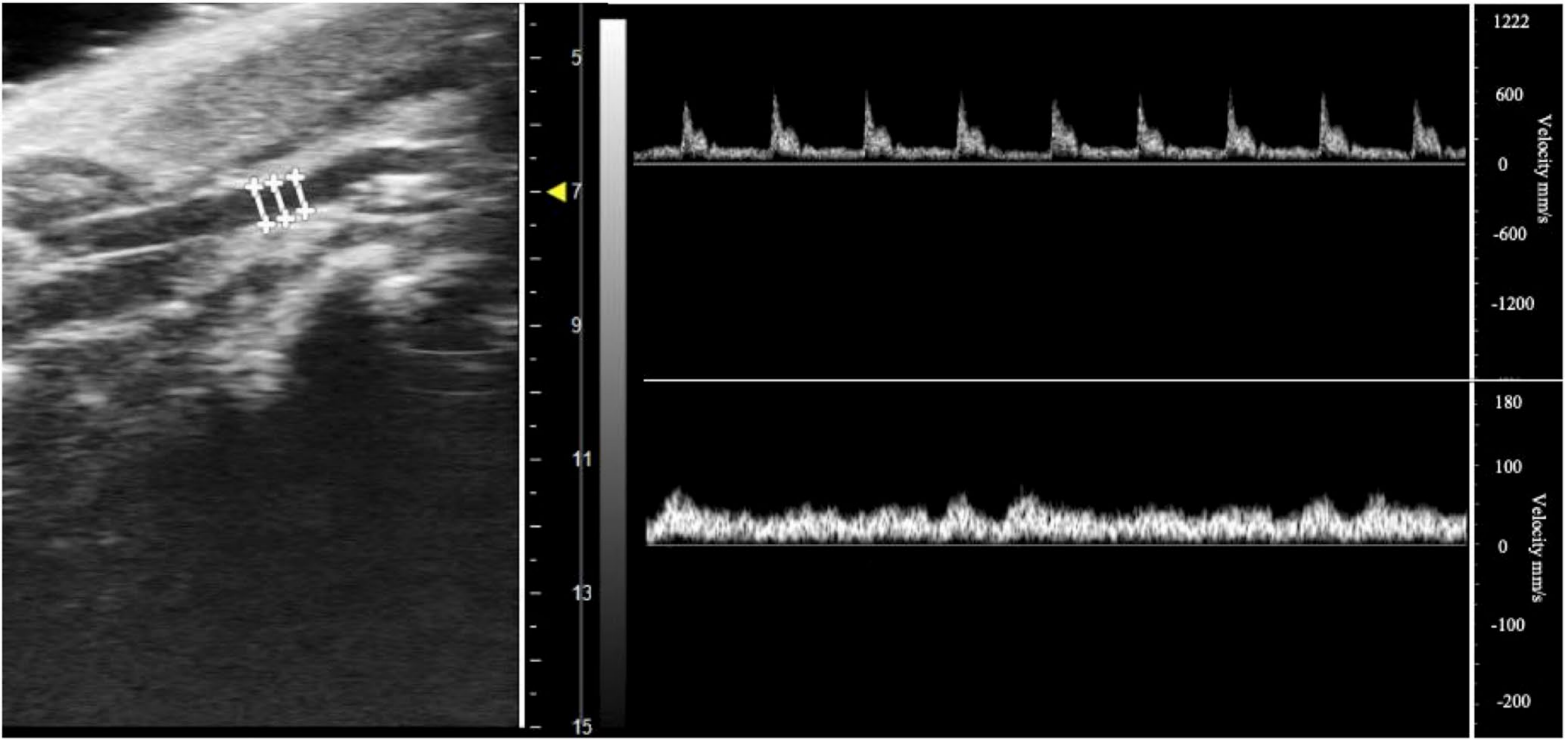
B‐mode image (left) and pulse waves of the carotid artery (top right) and jugular vein (bottom right) of a C57 mouse with diameter measurements shown. The average diameter of the artery was calculated for both systole and diastole. The diameter of the jugular vein was also measured at three different places across the cardiac cycle to provide an average diameter. For the carotid artery, three velocity peaks in systole across the cardiac cycle were measured and averaged to provide an average velocity and the process was repeated in diastole. For the jugular vein, three velocity measurements were averaged to provide an average velocity.

As shown in Figure 1, all mice were first imaged via ultrasound using the Vevo 3100 (Visual Sonics) with the MX550D: 55 MHz MX Series transducer. Images were collected of the carotid arteries and jugular veins: in B‐mode to measure the diameter and pulsed‐wave (PW) Doppler to measure velocity during systole and diastole (Figure 2). Pulsatility index– a non‐invasive method of measuring vascular resistance with higher values indicating greater pulsatile flow possibly indicating vascular stiffening (Wielicka et al., 2020)–was also calculated. VevoLab software (Visual Sonics) was used to analyze the ultrasound data.

Using the velocity and diameter for both the carotid in systole and diastole, the wall shear stress (WSS) was calculated. WSS was calculated using the following equation:

(1) $$\tau =\frac{8\mu \bar{u}}{d}$$

where μ is the viscosity of blood, 0.04 $\frac{g}{\mathrm{cm}\cdot s}$, u is the average velocity of blood, and d is the average diameter (Greve et al., 2006; Taylor et al., 1998; Wang et al., 2013). In previous studies, the Hagen‐Poiseuille application (Vlachopoulos et al., 2011) has been used for the WSS measurements in large arteries since these vessels mimic the structure of a cylindrical tube with rigid walls (Crouch et al., 2020; Wang et al., 2013).

For the carotid arteries, average volumetric blood flow, *V*_flow_, was calculated using the following equation,

(2) $$\begin{array}{c} V_{\text{flow}}=A\cdot V \end{array}$$

where A is the cross‐sectional area of the vessel and v is the average velocity of blood. Vascular flow was normalized by individual body weight to account for differences in blood volume for larger animals.

For the carotid arteries, the mean velocity was measured and used to calculate pulsatility index using the following equation:

(3) $$\begin{array}{c} \text{Pulsatility Index}=\frac{\mathrm{PSV}-\mathrm{EDV}}{\mathrm{MV}} \end{array}$$

where PSV is the peak systolic velocity, EDV is the end diastolic velocity, and MV is the mean velocity across the cardiac cycle (Bill et al., 2023).

The values of the diameters were used to calculate the circumferential cyclic strain (E) using the following equation,

(4) $$\begin{array}{c} E_{\theta \theta }=\frac{1}{2}\left(\frac{{D_{s}}^{2}}{{D_{d}}^{2}}-1\right) \end{array}$$

where $D_{s}$ represents the diameter during systole and $D_{d}$ represents the diameter during diastole (Crouch et al., 2018; Goergen et al., 2010; Humphrey & Delange, 2004).

#### 2.1.1 Statistical analysis

Using JMP, a one‐way ANOVA and a Tukey's Honest Significance Difference (HSD) post‐hoc test were performed to determine statistical difference between age groups for CCS, WSS in systole and diastole for the carotid, pulsatility index, and volumetric flow. Data is reported as mean and standard error for all data sets. *p*‐value was set to *p* < 0.05.

### 2.2 Tissue harvesting and preparation

After ultrasound, the mice were split into two groups, one for retrieval of the brains and the other for retrieval of the carotid arteries and jugular veins. For brain lipid analysis, young (*N* = 5 female) and middle‐aged (*N* = 5 female) mice were sacrificed via conscious decapitation for quick removal of the brain, and then brains were flash frozen on liquid nitrogen by floating the tissue in aluminum foil boats. The brains were cryosectioned at 10 μm and mounted on positively‐charged glass slides (Fisher) for MSI. Slides were stored in a −80° freezer until used for mass spectrometry. The location of each brain slice was determine using a standard mouse brain atlas (Allen Brain Atlas, 2025), and samples were collected at a location approximately −2.24 ± 0.21 mm from the bregma. For LC–MS analysis, brains were sectioned at 10 μm and six serial sections were collected in tubes.

For vascular MSI, the additional young (*N* = 5 female) and middle‐aged (*N* = 5 female) mice were sacrificed via isoflurane overdose followed by a thoracotomy to preserve the carotid arteries and jugular veins. Once the carotid, jugular, and brain were removed, the vessels were flash frozen on liquid nitrogen. The carotid and jugular vessels were embedded in Knox gelatin, cryosectioned at 10 μm, and mounted on slides using our previously published technique for blood clots (McDonald et al., 2023). The slides were stored in a −80° freezer until used for mass spectrometry.

### 2.3 Mass spectrometry

#### 2.3.1 MALDI‐MSI

For MALDI MSI analysis in positive ion mode, 2,5‐Dihydroxybenzoic acid (DHB) at 40 mg/mL in 70% MeOH was sprayed on the slides using an HTX M3+ sprayer. The matrix was sprayed on the tissue in a crisscross pattern 10 times at a temperature of 75°C and a pressure of 10 psi N_2_ gas. Sprayer parameters included flow rate of 100 μm/min, velocity of 1200 mm/min, track spacing of 3 mm, and a drying time of 10 s. For analysis in negative ion mode, 9‐Aminoacridine (9AA) at 10 mg/mL in 70% MeOH was sprayed on the tissue in a crisscross pattern 10 times at a temperature of 75°C and a pressure of 10 psi N_2_ gas. Sprayer parameters included flow rate of 100 μm/min, velocity of 1200 mm/min, track spacing of 2.5 mm, and a drying time of 0 s (McDonald et al., 2023). Once the tissue was sprayed with the matrix, slides were sent to Johns Hopkins AIMS core for MSI analysis. Slides were placed in a Bruker timsTOF fleX MALDI‐2 mass spectrometer and run in positive and negative mode with a pixel size set to 60 μm for brains and 10 μm for vessels (Crouch et al., 2020). Brain experiments were completed in both positive and negative ion mode with the following parameters: 60 micron single laser spot size, 60 micron raster, 200 laser shots per pixel, and an m/z range of 20 to 2000. Vessel experiments were completed in both positive and negative ion mode with the following parameters: 10 micron single laser spot size, 10 micron raster, 200 laser shots per pixel, and an m/z range of 20 to 2000. Imaging experiments were imported into SCiLS lab (Version 11.01.14,623) for analysis and normalized to total ion count (TIC).

#### 2.3.2 LC–MS

LC–MS was performed by the Biological and Small Molecule Mass Spectrometry Core (BSMMSC) at the University of Tennessee Knoxville to identify lipids and lipid‐like metabolites in the brain samples. Samples were stored at −80°C prior to extraction, which was conducted following the Bligh and Dyer method (Bligh & Dyer, 1959) using HPLC grade reagents (Thermo Fisher Scientific). Briefly, isotopically labeled internal standard (Avanti Research, Alabaster, AL) was added to the sample along with 750 μL of 1:2 chloroform: methanol and vortexed. Following mixing, 250 μL of chloroform and 250 μL of water were added; the solution was vortexed, and centrifuged for 5 min at 13,000 rpm. The lower layer (organic layer) was collected and added to a glass vial. The extraction was repeated with an additional 250 μL of chloroform and the resulting organic layer was pooled into the glass vial. Following vortexing, the extract was aliquoted and dried under a stream of nitrogen. LCMS analysis was performed using a Vanquish UHPLC coupled to an Exploris 120 mass spectrometer (Thermo Scientific, Waltham, MA). Lipids were separated on a Thermo Accucore C30 2.1 mm × 100 mm, 2.6 μm column using a gradient of mobile phase A (60:40 water: acetonitrile with 10 mM ammonium formate and 0.1% formic acid) and mobile phase B (90:10 isopropanol: acetonitrile with 10 mM ammonium formate and 0.1% formic acid) with the following gradient: 0 min 32% B; 1.5 min 32% B; 4 min 45% B; 5 min 52% B; 8 min 58% B; 11 min 66% B; 14 min 70% B; 18 min 75% B; 21 min 97% B; 25 min 97% B; 25.1 min 32% B; 30 min 32% B. The flow rate was set to 0.260 mL/min. Data were collected in both positive and negative mode using data dependent acquisition at a resolution of 120,000. Feature‐finding and lipid annotation were conducted using MS‐Dial (version 4.9) (Tsugawa et al., 2015). Raw area counts of annotated lipids in positive and negative mode were normalized by the internal standard and further processed using an in‐house Python script where normalized values were log transformed. Individual lipids annotated from the LCMS untargeted lipidomics analysis were cross‐referenced with those found from the MALDI MSI analysis for tentative lipid annotations for MALDI MSI. Lipid annotations and abbreviations can be found in Table 1.

**TABLE 1:** List of lipid classes and their respective abbreviations.

Abbreviation	Lipid class
AHexCer	Acetyl‐Hexosylceramide
CAR	Acylcarnitines
CER	Ceramides
CL	Cardiolipidins
DG	Diacylglycerols
FA	Free fatty acids
HexCer	Hexosylceramide
LPA	Lysophosphatidic acid
LPC	Lysophosphatidylcholines
LPE	Lysophosphatidylethanolamines
LPG	Lysophosphatidylglycerol
LPI	Lysophosphatidylinositol
LPS	Lipopolysaccharide
MGDG	Monogalactosyldiacylglycerols
NAE	*N*‐acylethanolamines
PA	Phosphatidic acids
PC	Phosphatidylcholines
PE	Phosphatidylethanolamines
PG	Phosphatidylglycerol
PI	Phosphatidylinositols
PS	Phosphatidylserines
SHexCer	Sulfated Hexosylceramide
SL	Saccharolipids
SM	Sphingomyelin
TG	Triacylglycerols

#### 2.3.3 Statistical analysis

Statistical changes in lipid headgroup composition were evaluated using normalized/log‐transformed LC–MS data from untargeted lipidomics. In each sample, lipids pertaining to a specific headgroup were summed and a *t*‐test (two‐tailed distribution, homoscedastic) was performed to evaluate statistical significance between two compared groups. Fold changes were also calculated to illustrate differential abundance of lipid headgroups for each comparison. MetaboAnalyst (version 5.0) (Pang et al., 2021) was used to perform a partial least squares discriminant analysis (PLSDA) on tested groups via one factor statistical analysis (Singam et al., 2019). For MALDI MSI, an unsupervised principal component analysis (PCA) was performed with SCiLS software to assess age‐related differences in brain and specifically, hippocampal lipid composition.

### 2.4 Staining

After the slides of carotid, jugular, and brain tissue were imaged via the MALDI MSI process, they were placed in a desiccator prior to staining. First, the matrix was removed using ethanol for 2 min and HPLC water for 3 min. After removing the matrix, the slide was left out to air dry for 5 min before beginning the staining protocol. A coverslip was placed on the slide to prepare for imaging with the Nikon Eclipse E600 microscope. The brain was stained using the Nissl staining protocol (StatLab, 2025). The carotid and jugular were imaged via elastin autofluorescence imaging and stained with Masson's Trichrome for collagen in the tissue (Electron Microscopy Sciences, 2025). Vessel images were captured on a Nikon Ti2 microscope and a W1 spinning disk confocal with a 488 nm laser at 50% power and 100 ms exposures on a Prime BSI Express camera. Images were visualized using Nikon Elements software. Elastin‐to‐collagen (E/C) ratios were quantified from histologically stained vascular sections using Fiji (ImageJ). Stained images were imported into Fiji, where color thresholding was applied to distinguish elastin‐positive and collagen‐positive regions. The area occupied by elastin and collagen was measured separately for each image, and the E/C ratio was calculated as the ratio of elastin area to collagen area. For each age group, measurements were averaged across multiple images (five images from young females and five images from middle‐aged females) to obtain representative elastin‐to‐collagen ratios for young and middle‐aged vessels.

### 2.5 Overall statistical analysis

Pearson correlation analysis was conducted in JMP to assess relationships between vascular biomechanical measures (CCS and WSS) and lipidomic variables, including lipid headgroup intensities obtained from LC–MS. Pairwise Pearson correlation coefficients were calculated, and correlations with |*R*| >0.6 were considered moderate to strong. Results were visualized using correlation matrices and heat maps.

## 3 RESULTS

### 3.1 Ultrasound

**FIGURE 3:**
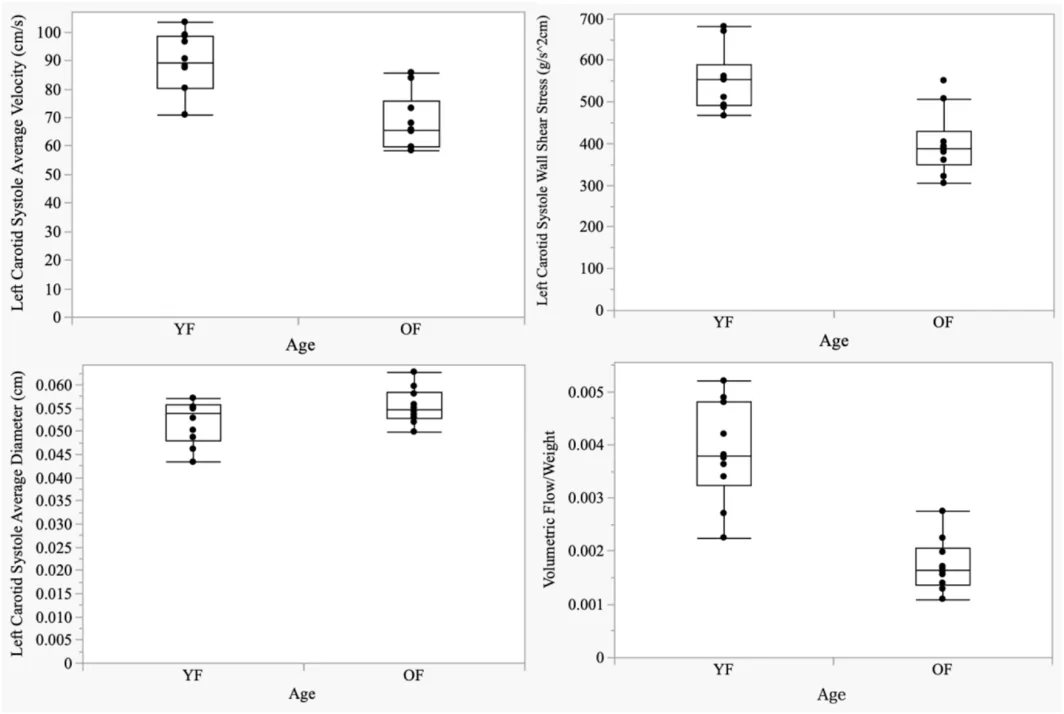
Effects of aging on left carotid artery hemodynamics and morphology in female C57BL/6 mice. Young female (YF) and middle‐aged female (OF) mice were compared for systolic average velocity, systolic wall shear stress, systolic average diameter, and volumetric flow normalized to body weight. Aging significantly decreased carotid systolic velocity, wall shear stress, and normalized volumetric flow, while increasing carotid diameter (*p < 0.05). Mean values reported on each graph. Box plots show the median (center line), interquartile range (box), and minimum‐to‐maximum values (whiskers); individual dots represent biological replicates.

Average carotid velocity during systole decreased by approximately 23.5% in middle‐aged females compared to young females (*p* = 0.0002). WSS in the common carotid artery during systole was significantly reduced with an approximate 27.8% decrease in middle‐aged females relative to young females (Figure 3, *p* = 0.0002). The reduction in carotid WSS during systole was driven primarily by the significant decline in average systolic velocity with aging. Average volumetric flow in the carotid artery, normalized to body weight, was also significantly reduced by ~55.2% with age (*p* < 0.0001).

The pulsatility index in the carotid artery showed a slight decrease of approximately 5.8% in middle‐aged females compared to young females; however, this difference was not statistically significant. These findings indicate that, despite age‐related trends toward reduced pulsatility, pulsatility index remained relatively preserved between young and middle‐aged females.

CCS in the carotid artery exhibited a slight decrease of approximately 3.9% in middle‐aged females compared to young females; however, this difference was not statistically significant. These results suggest that circumferential cyclic strain remains largely preserved with aging despite trends toward reduced vascular deformation.

The average jugular diameter was approximately 11.6% greater in middle‐aged females compared to young females with values of *p* = 0.0174.

### 3.2 Mass spectrometry

**FIGURE 4:**
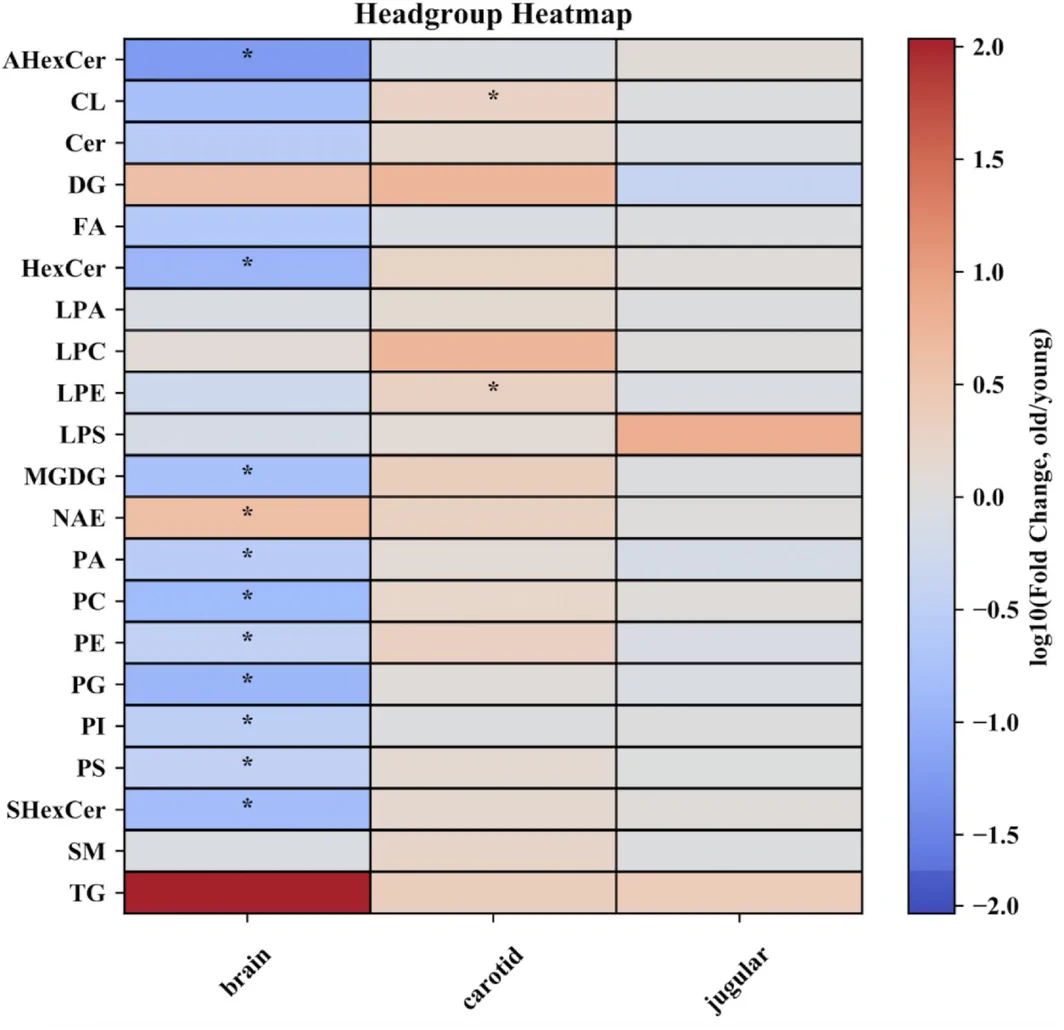
Heatmap illustrating age‐related changes in lipid headgroup abundances in the brain, carotid artery, and jugular vein of female mice. Values represent the log10 fold change of aged relative to young animals (old/young), with red indicating increased abundance and blue indicating decreased abundance with aging. Asterisks denote lipid headgroups that exhibited statistically significant differences between age groups (*p* < 0.05). This analysis highlights tissue‐specific lipid adaptations associated with aging.

**FIGURE 5:**
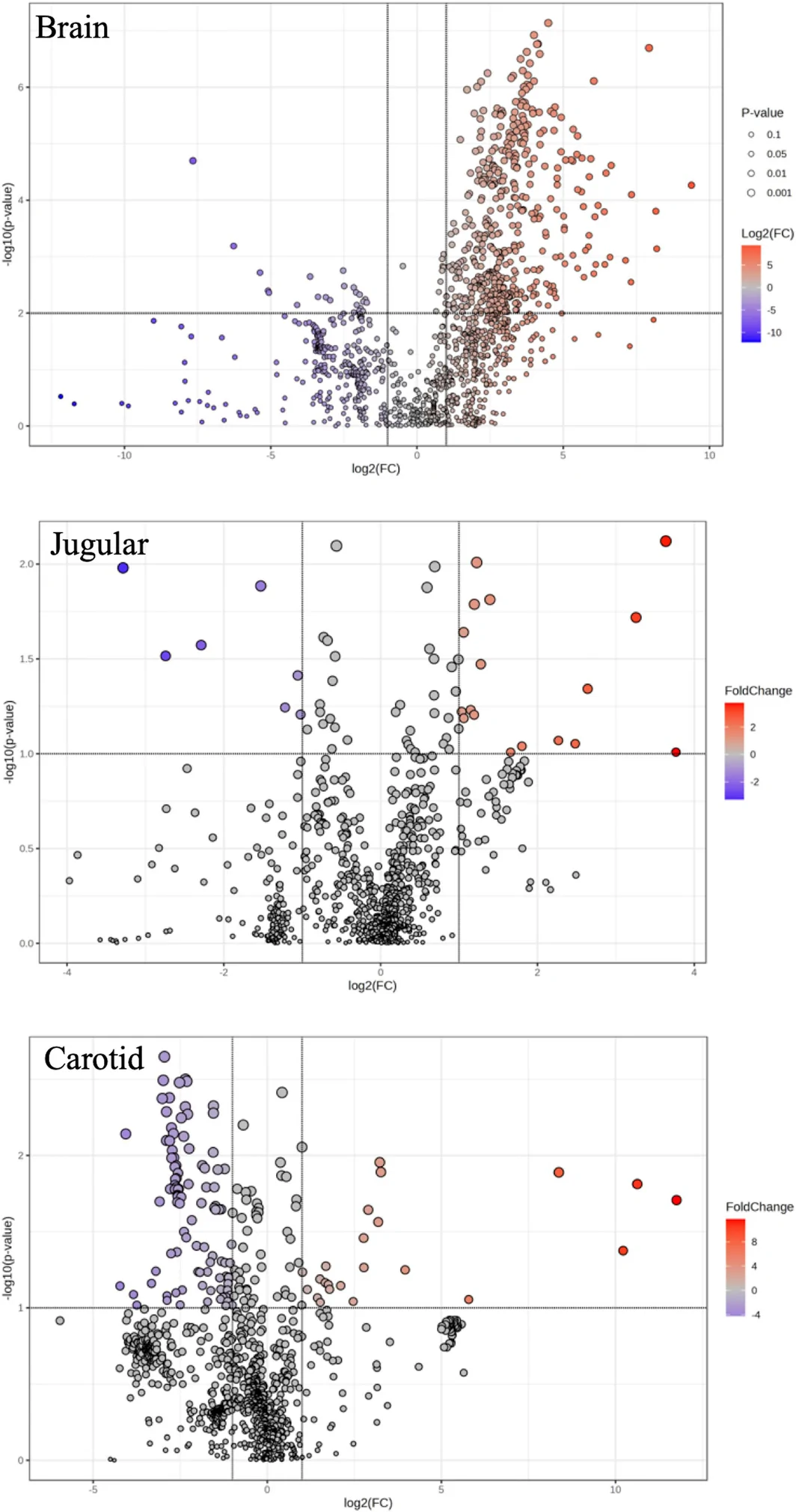
Volcano plot of lipids found within the brain, carotid artery, and jugular vein tissue of young (3 months) and old C57BL/6 females (12 months). The red indicates a higher abundance of a certain lipid in the younger population compared to blue which indicates a lower abundance in the younger population of female mice.

LC–MS results identified several lipid headgroups with significantly different fold changes between groups. Figure 4 depicts a list of the lipid headgroups found in all three tissue types and their respective fold change. In the brain, AHexCer, HexCer, MGDG, PA, PC, PE, PG, PI, PS, and SHexCer significantly decreased with respect to age. However, NAE significantly increased with respect to age. In the carotid, CL and LPE significantly increased with respect to age. Within each lipid headgroup, individual lipids contributed to the separation between young and middle‐aged females as seen in Figure 5. The spatial distribution of these lipids was further investigated using MALDI MSI.

**FIGURE 6:**
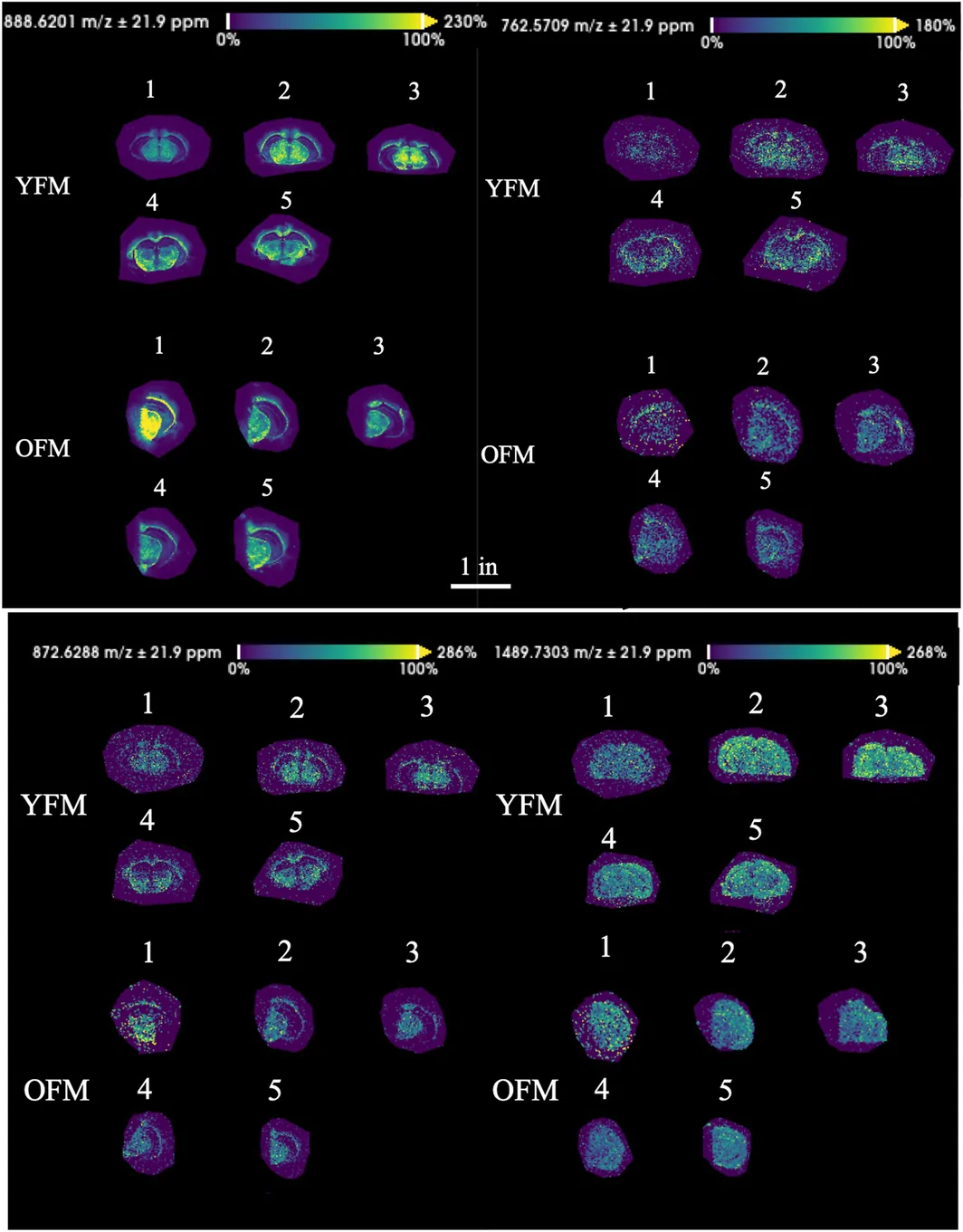
Top left: SHexCer 42:2 O2 with m/z of 888.62 present in the brain tissue of young and aged C57 females. The intensity decreased with age. Top right: HexCer 21:0;O2/12:0 with m/z of 762.56 in the young and aged C57 females. Bottom left: PS 42:1 with m/z of 872.63. Bottom right: Tentative cardiolipin species at m/z 1489.73.

MALDI MSI data revealed changes in the intensity and spatial distribution of several lipids with significant fold changes found from the LC–MS results. In the MALDI MSI and LC–MS analysis of the brain tissue, HexCer 21:0;O2/12:0 [M‐H]^−^, with m/z of 762.56, significantly decreased with respect to age with a fold change of 41.2 and spatial distribution depicted in Figure 6. SHexCer 42:2 O2 [M‐H]^−^, with m/z 888.62, had a higher intensity compared to young females as shown in Figure 6. PS 42:1 [M + H]^+^, with m/z of 872.63, significantly decreased with respect to age with a fold change of 35.4 and spatial distribution depicted in Figure 6. A lipid with m/z 1489.73, consistent with a cardiolipin‐associated species, decreased in signal intensity in the middle‐aged female hippocampus compared to young controls depicted in Figure 6.

**FIGURE 7:**
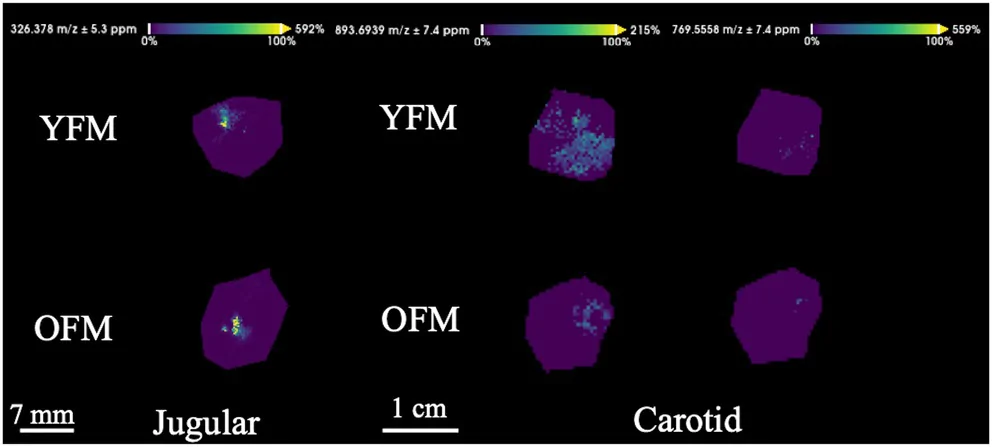
Left: An unknown lipid with m/z of 326.37 present in the jugular vein tissue of *top* young C57 female and *bottom* middle‐aged C57 female with a decrease in intensity with respect to age. Middle: DG 55:10 with m/z of 893.69 present in the carotid artery tissue of *top* young C57 female and *bottom* middle‐aged C57 female with a decrease in intensity with respect to age. Right: TG 45:9 with m/z of 769.54 present in the carotid artery tissue of *top* young C57 female and *bottom* middle‐aged C57 female with an increase in intensity with respect to age.

In the jugular vein, an unknown species, with m/z of 326.37 (tentative formula: C_21_H_46_N_2_), significantly decreased with respect to age with spatial distribution depicted in Figure 7 and a fold change of 13.5. In the carotid artery, TG 45:9, with m/z of 769.54 [M + H]^+^, was increased in middle‐aged females compared to young females, shown in Figure 7. DG 55:10, with m/z of 893.69 [M + H]^+^, decreased with respect to age in the carotid artery with a fold change of 8.06 and spatial distribution depicted in Figure 7. Figure S1 depicts intensity distributions for selected m/z features in brain, carotid, and jugular tissue from young and middle‐aged mice. The box plots highlight animal variability and suggest age‐associated differences in the abundance of several detected ions.

**FIGURE 8:**
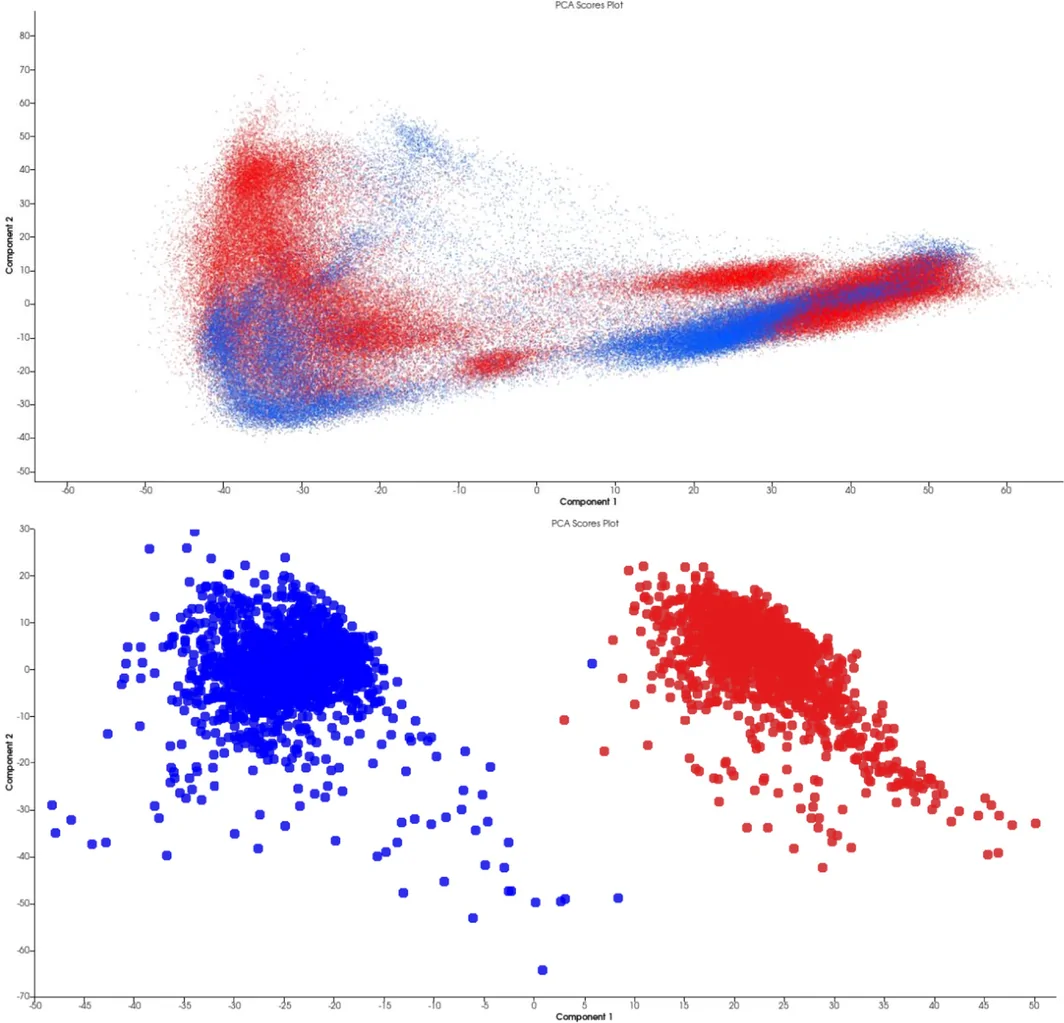
Unsupervised principal component analysis (PCA) of MALDI mass spectrometry imaging data from young and middle‐aged female mouse brains. Top: Whole brain PCA shows substantial overlap between age groups, indicating limited global separation of lipid profiles. Bottom: PCA restricted to hippocampal regions reveals distinct age‐dependent clustering with minimal overlap, demonstrating that lipid remodeling with aging is region‐specific and more pronounced within the hippocampus.

The PCA of whole‐brain spectra from our MALDI MSI data revealed substantial overlap between young and middle‐aged female mice, indicating limited overall separation of lipid profiles when analyzing the brain as a single heterogeneous structure as shown in Figure 8. Despite this overlap, a slight shift along the first principal component suggested underlying age‐associated lipid variation.

In contrast, the PCA restricted to hippocampal regions revealed a distinct separation between young and middle‐aged females along the first principal component, with minimal overlap between groups. This enhanced separation indicates that age‐dependent lipid remodeling is more pronounced within the hippocampus than at the whole‐brain level. The hippocampal PCA identified high‐mass lipid species as major contributors to group separation. These findings demonstrate that regional analysis reveals age‐related lipid alterations that are partially masked in overall brain analyses.

**FIGURE 9:**
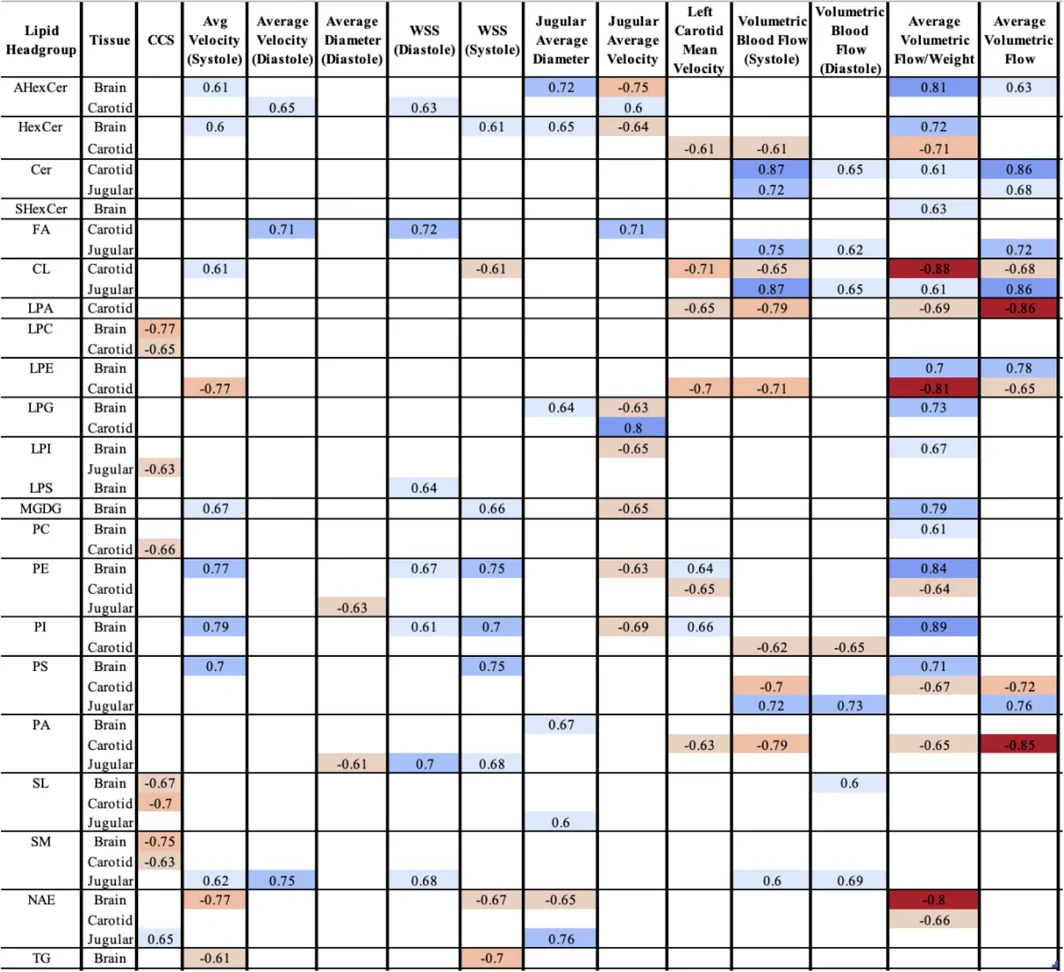
Heatmap showing Pearson correlation coefficients (r) between lipid headgroup intensities measured in brain, carotid, and jugular tissues and corresponding vascular biomechanical and hemodynamic parameters, including circumferential cyclic strain (CCS), velocity, diameter, wall shear stress (WSS), and volumetric flow metrics. Each cell represents the strength and direction of the linear relationship for a given lipid–parameter pair. Darker blue indicates stronger positive correlations, while darker red indicates stronger negative correlations; lighter shading reflects weaker associations. Blank cells denote correlations that were not significant or not available.

**FIGURE 10:**
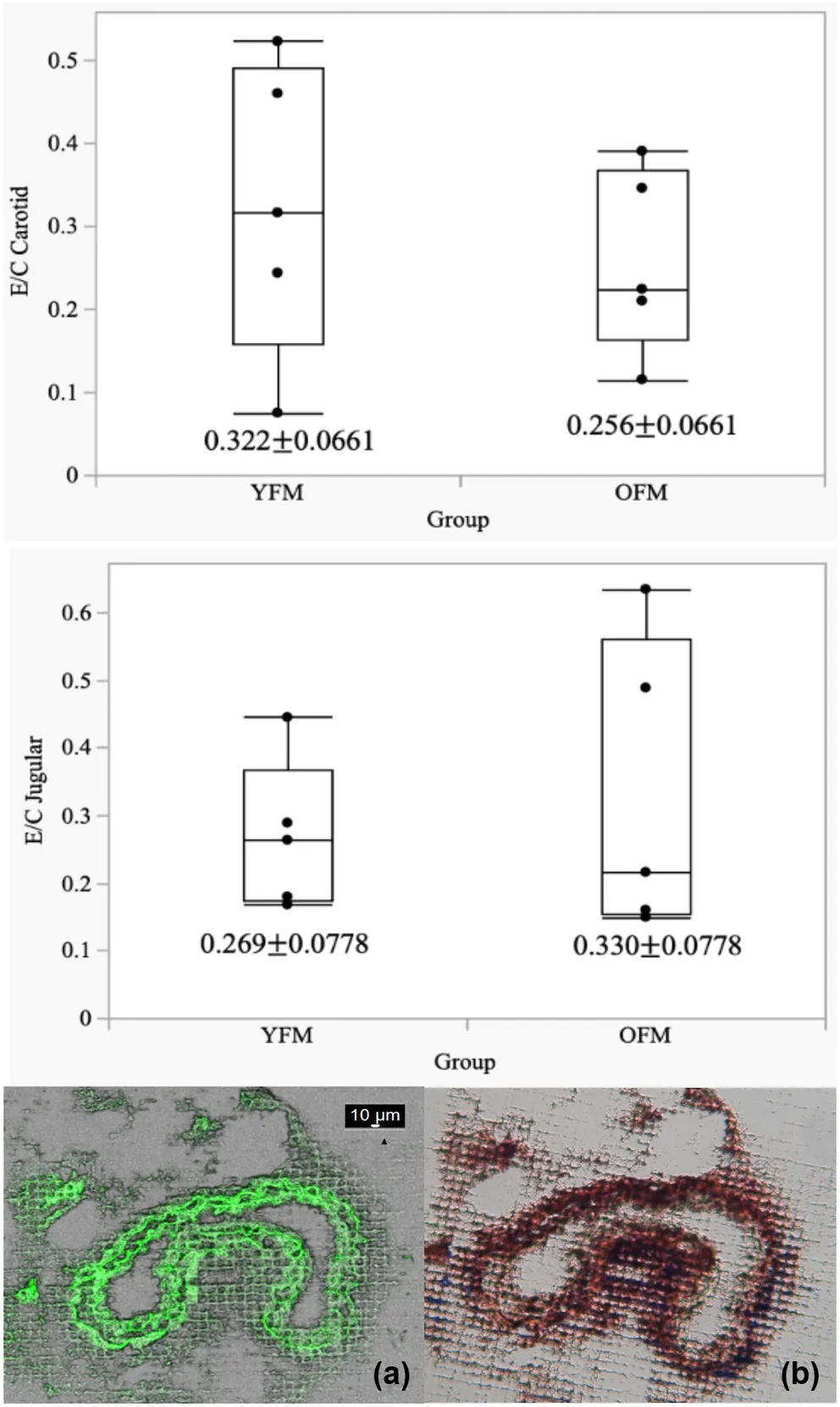
Top: Quantitative analysis of elastin‐to‐collagen (E/C) ratio in carotid arteries from young female mice (YFM) and middle‐aged female mice (OFM). Individual dots represent independent animals. Boxes indicate the interquartile range, and the center line indicates the median. Mean values reported on each graph. Middle‐aged mice exhibited a lower average carotid E/C ratio, consistent with age‐related vascular remodeling. Middle: Quantitative analysis of elastin‐to‐collagen (E/C) ratio in jugular veins from young female mice (YFM) and middle‐aged female mice (OFM). Bottom: (a) Carotid artery middle‐aged C57 mouse imaged with elastin autofluorescence (b) Stained with Masson's trichrome. Collagen is shown in blue, muscle/cytoplasmic tissue in red/pink, and nuclei in dark purple/black.

Pearson correlation analysis revealed distinct relationships between vascular biomechanical parameters and lipid features across tissues. Several lipid headgroups and m/z features exhibited moderate to strong correlations (|*R*| >0.6) with carotid CCS and WSS, indicating coordinated associations between vascular function and lipid composition (Figure 9). Both positive and negative correlations were observed, highlighting lipid‐specific associations with vascular biomechanics. Figure 10 shows the quantitative analysis of the E/C ratio, the stained images, and autofluorescent images of the carotid artery of a middle‐aged C57 female. The area of elastin and collagen was computed and used to obtain the E/C ratio in the vascular tissue. The E/C ratio showed a lower mean value in carotid arteries from middle‐aged mice (OFM) relative to young female mice (YFM) (0.256 ± 0.0661 vs. 0.322 ± 0.0661), suggesting age‐related extracellular matrix remodeling in the carotid wall. In the jugular vein, the mean E/C ratio was higher in OFM than YFM (0.330 ± 0.0778 vs. 0.269 ± 0.0778). These data indicate potentially distinct age‐related patterns of structural remodeling in arterial and venous neck vessels.

## 4 DISCUSSION

### 4.1 Aging resulted in significant changes in hemodynamics and lipid, elastin, and collagen composition of neck vasculature

The average values of WSS for the carotid artery decreased with age, which aligns with the results of other research groups (Crouch et al., 2020; Kamimura et al., 2023; Samijo et al., 1998). In addition, human studies have consistently shown that average carotid WSS decreases with age along with increased arterial stiffness (Irace et al., 2012). The decrease seen in WSS was driven by a significant decrease in PSV. The observed decrease in PSV may be attributed to age‐related changes in vascular compliance and cardiac output, contributing to a decrease in hemodynamic forces acting on the vessel wall (Mahmoud, 2018; Singam et al., 2019). These hemodynamic forces can result in vascular wall remodeling leading to changes in CCS and pulsatility index (Davies, 2008). Although the values of CCS and pulsatility index in our current study did not exhibit statistical significance between age groups, the average values of CCS decreased with age, consistent with previous studies (Lin et al., 2008). This age‐related reduction in CCS may indicate increased arterial stiffness.

In the present study, an age‐related decrease in systolic velocity and WSS was accompanied by significant changes in intensity and spatial distribution of vascular lipid species, including alterations in glycerolipids and sphingolipids in the carotid artery and jugular vein. Since membrane lipids, such as glycerolipids and sphingolipids, play an important role in membrane structure, mechanosensing, and endothelial responses to shear stress, these findings suggest that age‐related lipid remodeling may contribute to changes in vascular function (Donato et al., 2009; Li & Kim, 2021). Changes in glycerolipids suggest shifts in membrane composition and lipid signaling that are important for maintaining endothelial barrier function and responding to blood flow. Glycerolipids can also act as sources of fatty acids that influence inflammation and oxidative stress, both of which are linked to vascular aging (Van Meer et al., 2008). Changes in diacylglycerol DG levels may affect vasomotor control, mechanosensing, and how blood vessels respond to shear stress (Rask‐Madsen & King, 2007). A significant age‐related decrease in DG 55:10 in the carotid artery may reflect a decrease in lipid signaling potentially contributing to impaired endothelial responsiveness and vascular adaptability with aging (Liu et al., 2024). In contrast, sphingolipids play key structural and signaling roles in vascular membranes. Changes in sphingolipid levels may therefore affect nitric oxide signaling, inflammatory pathways, and vascular compliance (Hannun & Obeid, 2018). Prior work has demonstrated that aging in females is associated with alterations in endothelial pathways, which contributes to increased arterial stiffness (Lekontseva et al., 2010). These findings suggest that changes in vascular lipid composition may work alongside age‐related endothelial dysfunction to drive changes in vascular mechanics with aging, even in the absence of disease‐related pathology.

Research indicates that triglycerides (TG) are a standard blood lipid with the strongest connection to arterial stiffness (Baba et al., 2023). In the carotid artery, TG 45:9 significantly increased with respect to age. Blood lipid biomarkers are indicative of arterial stiffness, such as standard blood lipids, non‐conventional lipid markers, and lipid ratios (Baba et al., 2023); however, other classes of lipids in the vessel walls have not been thoroughly investigated. Other research investigating lipid composition within the vasculature has largely focused on pathological conditions, particularly atherosclerosis. Atherosclerosis, a type of vascular dysfunction, causes plaque formation in the artery walls leading to thickening and hardening of the arteries (Moerman et al., 2021). The plaque buildup is mainly caused by the accumulation of lipids. The increase in lipids and the resulting modification in the vessel wall causes an inflammatory response. In the carotid artery, CL and LPE levels increased with age, potentially reflecting changes in mitochondrial function and membrane remodeling within the vascular wall (Mueller et al., 2015; Paradies et al., 2014). Changes in the abundance of CL have been linked to vascular dysfunction and an age‐related decrease in mitochondrial efficiency (Chicco & Sparagna, 2007). Similarly, changes in the abundance of LPE have been linked to endothelial activation, changes in membrane fluidity, and vascular remodeling with aging (Hirata et al., 2021; Xu et al., 2024). These findings suggest that aging leads to tissue‐specific changes in lipids and that vascular dysfunction may occur alongside biochemical changes in vascular tissue.

Histological analysis of the carotid artery further revealed age‐related structural remodeling of the vascular wall not captured by ultrasound imaging. Analysis of stained sections revealed a decrease in the elastin‐to‐collagen ratio in middle‐aged female mice compared to young controls. Elastin provides arterial elasticity and recoil, whereas collagen contributes to stiffness; therefore, a reduced elastin‐to‐collagen ratio is indicative of increased arterial stiffness with aging (Trębacz & Barzycka, 2023). The observed decrease in the elastin‐to‐collagen ratio likely reflects diminished arterial compliance, which may limit the vessel's ability to adapt to changes in pulsatile blood flow. This loss of compliance may underlie the age‐associated reductions in peak systolic velocity and wall shear stress observed in this study. The histological and functional findings suggest that aging leads to both structural and hemodynamic changes in the carotid artery. Although the carotid elastin‐to‐collagen ratio was lower in middle‐aged female mice than in young females, the jugular vein showed a modest increase, suggesting that aging may differentially affect extracellular matrix remodeling in arterial and venous neck vessels. Arterial stiffness can contribute to reduced flow and altered hemodynamic regulation. These hemodynamic changes can contribute to reduced blood flow and disrupted flow regulation. The average volumetric flow, normalized to body weight, decreased with age. Normalization to body weight suggests that this decrease reflects intrinsic changes in vascular function rather than differences due to body mass. This reduction in volumetric flow normalized to body weight indicates decreased blood volume to the brain. Age‐related changes in neck vasculature can also contribute to alterations in brain biochemistry (Jones et al., 2025).

### 4.2 Aging is associated with substantial remodeling of lipid species in the brain

Age‐related alterations in lipid composition were observed across the brain tissues, indicating tissue‐specific biochemical remodeling with aging. The decrease in these lipid headgroups, including AHexCer, HexCer, MGDG, PA, PC, PE, PG, PI, PS, and SHexCer, indicates changes in membrane integrity, myelination, and cellular signaling (Blusztajn et al., 2023; Choudhary et al., 2024; Lipotype, 2026). The observed decrease may reflect altered neuronal and glial function associated with aging (Donato et al., 2015; Donato et al., 2018). In contrast, NAEs were significantly increased with age, which may represent a compensatory response to age‐related stress or inflammation, as NAEs have been implicated in neuroprotective and neuromodulatory processes (Rappley et al., 2009). In a study using 3–4 month, 10 month, and 19–21 month C57Bl6 male mice, PC and HexCer increased with respect to age (Panda et al., 2025). Another study using 3–4 month mice and 12–14 month mice revealed a decrease in levels of PS, PG, PI, and PC, but an increase in PA with age (Rappley et al., 2009). This same study investigated whether lipid composition was sex‐dependent in wild‐type mice and found that there were no obvious differences but did notice more age‐dependent variability in males than females. In females, PG, PI and PS decreased aligning with our results (Rappley et al., 2009). Analyzing different strains revealed age‐related changes are specific to different genetic backgrounds (Rappley et al., 2009).

MALDI MSI analysis complemented the LC–MS findings by revealing age‐related changes in both the intensity and spatial distribution of several lipid species in the brain tissue. HexCer 21:0;O2/12:0 (m/z 762.56) showed region‐specific reductions in the brain, which may reflect alterations in sphingolipid metabolism and myelin integrity. In contrast, SHexCer 42:2;O2 (m/z 888.62) was more abundant in middle‐aged females, suggesting an accumulation of sulfated hexosylceramides with age (Han et al., 2002). SHexCer 42:2;O2 has also been shown to increase with age in male mice from the ages of 3 month through 21 months (Panda et al., 2025). Additionally, PS 42:1 (m/z 872.63) levels were lower in middle‐aged brain tissue, indicating potential age‐related changes in membrane composition and phospholipid signaling (Vance & Tasseva, 2013).

### 4.3 Bulk lipidomic profiling of the brain tissue fails to capture significant region‐specific lipid alterations

The hippocampus exhibits substantially greater biochemical differences than the brain as a whole. While whole‐brain PCA showed limited separation between young and middle‐aged samples, hippocampus analysis revealed age‐dependent clustering, emphasizing the importance of region‐focused investigation in studies of brain aging (Naudí et al., 2015). A major contributor to hippocampal separation is a cardiolipin‐associated ion at m/z 1489.73. Cardiolipins are essential components of mitochondrial membranes and play a critical role in maintaining mitochondrial structure and function (Chicco & Sparagna, 2007; Fuentes & Morcillo, 2024). The observed decrease in this cardiolipin‐associated lipid in the middle‐aged female hippocampus is consistent with established models of mitochondrial dysfunction in brain aging. Importantly, the increased separation observed in hippocampal PCA compared to whole‐brain analysis highlights how regional susceptibility may underlie cognitive and functional decline with aging. The hippocampus is known to be particularly sensitive to metabolic and mitochondrial stress, and region‐specific lipid alterations may contribute to its heightened vulnerability (Small et al., 2002; Söderberg et al., 1990). By combining MALDI MSI with multivariate analysis, these results suggest that age‐related lipid changes occur in specific brain regions rather than uniformly across the brain.

### 4.4 Age‐related cardiovascular metrics correlate with vascular and brain lipidomics

Moderate to strong correlations between CCS and WSS and specific lipid classes, including glycerolipids such as DGs and TG‐associated species, phospholipids such as CL and LPE, and sphingolipids, suggest that age‐related changes in vascular function are closely associated with alterations in lipid composition within vascular and brain tissues. Although these correlations do not imply causality, they highlight interconnected structural and biochemical changes that occur with vascular aging. These findings support the concept that lipid remodeling may contribute to, or reflect, functional changes in vascular mechanics during aging and provide a foundation for future mechanistic studies examining the role of specific lipid pathways in neurovascular dysfunction. Because neuronal, glial, and brain functional endpoints were not directly measured in the present study, any links between the observed vascular and lipid changes and downstream neurobiological consequences should be considered speculative. In addition, comparisons with prior aging studies should be interpreted in the context of animal age, as our 52‐week‐old mice represent a middle‐aged cohort and may not fully recapitulate changes reported in substantially older animals.

### 4.5 Limitations

One limitation of this study is the challenge of ensuring that imaging and biochemical data were acquired from identical anatomical locations across mice. To mitigate this variability, consistent anatomical landmarks were used, with the carotid and jugular bifurcation serving as reference points for vascular measurements. While this approach improves spatial consistency, minor positional variability cannot be completely excluded. Although several biomechanical parameters differed significantly between groups, the effect sizes were relatively small; therefore, these findings should be interpreted cautiously as evidence of subtle vascular remodeling rather than large physiologically disruptive changes in cerebral hemodynamic regulation. Additionally, the selected age groups, approximately 12 weeks for young mice and 52 weeks for middle‐aged mice, were chosen based on prior studies; however, these time points may not fully capture the progression of vascular and biochemical aging. Intermediate and later age groups, such as 6 months for young adults and 18–20 months for aged mice, which more closely correspond to human ages of approximately 30 and 56–60 years, respectively (Dutta & Sengupta, 2016), may provide further insight into age‐dependent changes. We hypothesize that more advanced aging or the development of pathological conditions may be required to observe significant increases in vascular metrics, and it is possible that the middle‐aged mice used in this study had not yet reached a senescent stage. Future studies incorporating additional age groups could help refine the temporal relationship between vascular dysfunction and brain biochemical alterations.

## 5 CONCLUSION

This study demonstrates that aging is associated with functional, structural, and biochemical changes within the neurovascular system of female mice, even in the absence of disease. Age‐related reductions in volumetric blood flow, peak systolic velocity, and wall shear stress in the carotid artery indicate a decline in vascular function and hemodynamic regulation with age. These functional changes were accompanied by evidence of increased arterial stiffness, including decreased carotid compliance and a reduced elastin‐to‐collagen ratio, supporting the presence of vascular remodeling that limits adaptability to pulsatile flow.

Integration of LC–MS lipid profiling with MALDI MSI provided spatially resolved insight into these biochemical changes, revealing region‐specific lipid alterations within the brain, including the hippocampus, and localized lipid remodeling within the carotid artery and jugular vein.

These findings establish a foundational framework linking age‐related vascular dysfunction to biochemical changes in the brain. This work provides critical baseline data for future studies in pathological models, such as neurodegenerative disease, and highlights the importance of considering vascular‐biochemical interactions in the study of brain aging. Additionally, sex may be a factor for differences in incidence rate and progression of disease for both arterial stiffening and neurological diseases which emphasizes the need for further research in this area (Merz & Cheng, 2016).

## AUTHOR CONTRIBUTIONS

**Allison R. Jones:** Conceptualization; data curation; formal analysis; investigation; methodology; project administration; supervision. **Amin Jarrahi:** Data curation; formal analysis. **Kylee Karpowich:** Data curation; formal analysis. **EllaGrey Moody:** Data curation; formal analysis. **Lily Renner:** Data curation; formal analysis. **Lindsay P. Brown:** Data curation; formal analysis. **Caitlin M. Tressler:** Data curation; formal analysis; investigation. **A. Colleen Crouch:** Conceptualization; funding acquisition; project administration; resources; supervision.

## FUNDING

We would like to acknowledge NIH S10 OD030500 which funded the Bruker timsTOF fleX MALDI‐2 instrument at Johns Hopkins Applied Imaging Mass Spectrometry Core and the University of Tennessee Advanced Microscopy and Imaging Center (RRID:SCR_028273) for instrument use and scientific and technical assistance.

## Supporting information


**Figure S1.** Representative SCiLS Lab intensity distributions are shown for the annotated m/z values indicated on each panel. Box plots display ion intensity across individual brain samples from young female mice (YFM1–YFM5) and middle‐aged female mice (OFM1–OFM5). The center line indicates the median, the box represents the interquartile range, and whiskers show the spread of the data. These plots illustrate sample‐to‐sample variability and age‐related differences in the abundance of selected brain lipid‐associated ions.

## Data Availability

All data associated with this study are available in the Dryad Digital Repository. Additional information is available from the corresponding author upon reasonable request.
